# Alleviation of temporomandibular joint osteoarthritis by targeting RIPK1‐mediated inflammatory signalling

**DOI:** 10.1111/jcmm.17929

**Published:** 2023-08-29

**Authors:** Xin Cao, Sisi Peng, Ying Yan, Jun Li, Jianping Zhou, Hongwei Dai, Jie Xu

**Affiliations:** ^1^ College of Stomatology, Chongqing Medical University Chongqing China; ^2^ Chongqing Key Laboratory of Oral Diseases and Biomedical Sciences Chongqing China; ^3^ Chongqing Municipal Key Laboratory of Oral Biomedical Engineering of Higher Education Chongqing China

**Keywords:** Caspase‐8, cIAP, MLKL, RIPK1, RIPK3, TNF‐α

## Abstract

Temporomandibular joint osteoarthritis (TMJOA), prevalent in adolescents and the elderly, has serious physical and psychological consequences. TMJOA is a degenerative disease of the cartilage and bone, mostly driven by inflammation, and synoviocytes are the first and most important inflammatory factor releasers. Receptor‐interacting serine/threonine‐protein kinase (RIPK1) promotes inflammatory response and cell death during an array of illnesses. This research aimed to explore the impacts of RIPK1 inhibitor therapy in TMJOA and the mechanism of RIPK1 in inducing inflammation during TMJOA. Herein, inhibition of RIPK1 suppressed the elevated levels of inflammatory factors, nuclear factor kappa B (NF‐κB), along with markers of apoptosis and necroptosis after tumour necrosis factor (TNF)‐α/cycloheximide (CHX) treatment in synoviocytes. Moreover, inflammation models were constructed in vivo through complete Freund's adjuvant (CFA) induction and disc perforation, and the findings supported that RIPK1 inhibition protected TMJ articular cartilage against progressive degradation. RIPK1 regulates NF‐κB activation via cellular inhibitor of apoptosis proteins (cIAP), apoptosis via caspase‐8, and necroptosis via RIPK3/mixed lineage kinase domain‐like (MLKL) in synoviocytes, which in turn facilitates TMJOA inflammation progression.

## 1 INTRODUCTION

Temporomandibular joint (TMJ) is often attacked by osteoarthritis.^1^ Temporomandibular joint osteoarthritis (TMJOA) may lead to synovial inflammation, cartilage degradation, and subchondral bone reconstruction, causing symptoms such as pain, biting discomfort, or restricted mouth opening, all of which can have serious physical and psychological impacts on patients.^2^, ^3^ TMJOA has a high incidence, more prevalent among adolescents and elderly people.^4^ The fibrocartilage of the condyle makes TMJ different from other joints in terms of structure and growth.^5^ Current treatment of TMJOA aims at relieving symptoms and delaying the progression of the disease, but the results are not satisfactory.^6^ There is a need to study the pathogenesis and develop new treatments for the cause of the disease.

Recent evidence demonstrates that synovial inflammation can induce TMJOA.^7^ Invaded by inflammation, synoviocytes lining the synovial membrane undergo pathology that changes synovial fluid composition and joint function triggering symptoms of TMJOA.^8^ Inflammation‐activated synoviocytes secrete chemokines to attract immunocytes and proinflammatory cytokines, a mechanism that facilitates angiogenesis and extracellular matrix (ECM) degradation.^9^ Increasing studies indicate that synovial fibroblast‐mediated inflammation can be manipulated to control arthritic degeneration.^10^, ^11^ With the overproduction of pro‐inflammatory signals, like interleukin (IL)‐1β, and TNF‐α in the synovium, cartilage, or bone, there is a high level of matrix‐degrading proteases, mainly matrix metalloproteinases (MMPs) and A Disintegrin and Metalloproteinase with Thrombospondin Motifs (ADAMTs).^12^ Therefore, new strategies for treating TMJOA should be designed that target synoviocyte‐mediated inflammation in order to limit inflammatory progression at an early stage.^13^

RIPK1 regulates a series of inflammatory signalling pathways including Tumour necrosis factor receptor 1 (TNFR1), IL‐1, Fas ligands, necrosis, apoptosis and others.^14^ As an important inflammatory signalling regulator, it promotes cell proliferation, apoptosis and necroptosis.^15^ As a scaffolding molecule, RIPK1 promotes inflammatory gene transcription by activating the mitogen‐activated protein kinases (MAPK) and NF‐κB pathways.^16^ RIPK1 is also an active kinase that can drive apoptosis or necrosis, based on the intracellular environment.^17^ TMJOA occurs when large amounts of inflammatory molecules are present in the joint cavity. Therefore, we hypothesize that these inflammatory factors could converge with RIPK1 and activate downstream NF‐κB, caspase‐8/MLKL, etc., regulating cellular inflammatory cytokines release, apoptosis, or necroptosis. These manifestations of TMJOA synoviocytes may be related to the regulatory mechanism of RIPK1. Necrostatin‐1 (Nec‐1) is a targeted inhibitor that blocks the phosphorylation of RIPK1, thereby interfering with signalling pathways associated with the regulatory role of RIPK1.^18^ However, one study found that inhibition of RIPK1 also silenced NF‐κB and attenuated the generation of inflammatory factors in cisplatin‐induced nephrotoxic mice.^19^ Another study also detected apoptosis and necrosis in synoviocytes following simultaneous exposure in TNF‐α and CHX in vitro.^14^ This suggests that RIPK1 may play an important regulatory role in synoviocyte death.

In the past 2 years, several studies have confirmed that RIPK1 is a significant inflammatory signalling regulator.^20^ Currently, the targeted therapy of RIPK1 is highly valued internationally.^21^ However, little research has been done on RIPK1 in TMJOA and its contribution to the development of TMJOA is not widely recognized. Therefore, this study focuses on exploring the regulatory mechanism of RIPK1 in TMJOA and verifying the therapeutic effects of its inhibitor, in order to propose new directions for osteoarthritis treatment in clinics.

## 2 METHODS AND MATERIALS

### 2.1 Rat model of TMJ inflammation

Chengdu Dashuo Experimental Animal Co., Ltd. provided Sprague–Dawley (SD) rats. A total of 30 SD rats (350 g, ages 10 weeks) were grouped into TMJOA models induced using CFA (CFA, Sigma‐Aldrich) or disc perforation. Each model had a sham group (*n* = 5), a TMJOA model group (*n* = 5), and a treatment group (*n* = 5). CFA (50 μL) was given as an injection into the upper compartment of the bilateral TMJs to induce inflammation at day 0. The same amount of saline was injected into the control group. Meanwhile, the disc was perforated to establish the other TMJOA model. In detail, the zygomatic arch was incised in an oblique manner. Then, after exposure to TMJ superior joint space, the disc was dragged out, then drilled by a ball‐shaped drill, till a hole (1.5 mm in diameter) was made in the centre of the TMJ disc. However, this surgery was not performed in sham rats. The next day, the groups were injected with either 50 μL Nec‐1 (1 mg/kg) or 50 μL phosphate‐buffered solution (PBS) into the bilateral TMJ. The Nec‐1/PBS was injected again on day 4. Postoperative complications were not observed. All SD rats were sacrificed at 1 week for further studies. All rats were euthanized by CO2 overdose inhalation. Animal results were reported according to the ARRIVE guidelines.

### 2.2 Histological staining

TMJ specimens were fixed in 4% paraformaldehyde and then decalcified in 10% EDTA for 3 months. After decalcification, the tissues were dehydrated, embedded in paraffin and sectioned (5 μm thick). The orientation of slices in rat TMJ specimens was sagittal plane orientation. The tissue sections were then stained with haematoxylin and eosin (H&E) (Solarbio), sarfarin O and fast green (Solarbio). The morphology of the cartilage and synovium were scored using the Osteoarthritis Research Society International (OARSI) and arbitrary scales, respectively.^22^, ^23^

### 2.3 Immunohistochemistry

RIPK1 (1:100, Affinity Biosciences), RIPK3 (1:100, Bioss), MLKL (1:100, Immuno way), Caspase‐8 (1:100, Bioss), MMP1 (1:100, Bioss), MMP3 (1:100, Bioss) and MMP9 (1:100, Bioss) were immunohistochemically stained. Specimens were treated with first antibodies overnight at 4°C, rinsed in PBS and then immersed in secondary antibody treatment with horseradish peroxidase conjugate for 15 min. PBS was supplemented with negative controls. Diaminobenzidine was used to develop colour. After counterstaining with haematoxylin, the samples were placed on an Olympus microscope (Olympus) for observation and photography.^24^

### 2.4 Sample collection and cell culture

TMJ synovium and cartilage were collected from 15 individuals (seven males and eight females, average ages 28.3 yrs, range 21–35 yrs) receiving high condylectomy in the Stomatological Hospital of Chongqing Medical University for condylar fracture. These samples were considered normal when no synovial inflammation, synovial hyperplasia and cartilage inflammation were histologically confirmed by at least two pathologists.

Normal synovium samples were rinsed with PBS, then cultured (primary culture of 1 mm^3^) in dulbecco's modified eagle medium (DMEM)/F12 containing 10% foetal bovine serum (FBS), 1% antibiotics (streptomycin sulfate, and penicillin) (37°C, 5% CO2). The synoviocytes were allowed to free from the synovium. The separated synoviocytes in passages 3 and 5 were subjected to subsequent experiments, and synoviocytes were used to construct a homogenous population.

The cartilage was sectioned into pieces sizing 3 mm^2^ and cultured in DMEM/F12 plus 3 mg/mL collagenase I (37°C, 1 h). The chondrocytes were allowed to free from the cartilage. The chondrocytes were rinsed, reconstituted in DMEM/F12, and cultured. There times per week, the medium was replaced.

### 2.5 Cell viability assay

The effect of Nec‐1 on synoviocyte viability was assessed using the Cell Counting Kit‐8 (CCK‐8; BIOSS). Briefly, primary synoviocytes were planted into 96‐well plates (10^4^ cells/well), and then stimulated with Nec‐1 (0, 20, 40 and 60 μM for 6, 12 or 24 h). In addition, synoviocytes were subjected to TNF‐α (10 or 100 ng/mL), and simultaneously sensitized with CHX (5 or 10 μg/mL). After discarding the culture, 100 μL DMEM/F12 containing 10% CCK‐8 was added. After 4 h of cell culture at 37°C, synoviocyte viability was checked at 450 nm by a microplate reader (Bio‐Rad).

### 2.6 Cell treatment and grouping

The TMJ synoviocytes were assigned to control (N), TNF‐α + CHX group (TC, induction by 10 ng/mL TNF‐α [Sino Biological] and 10 μg/mL cycloheximide [CHX, Abmole]), TNF‐α + CHX + SM164 group (TCS, induction by 10 ng/mL TNF‐α, 10 μg/mL CHX, and 10 ng/mL SM164 [Medchemexpress]; synoviocytes were challenged with 10 ng/mL SM164 for 2 h before induction with TNF‐α + CHX), TNF‐α + CHX + Nec‐1 group (TCN, induction by 10 ng/mL TNF‐α, 10 μg/mL of CHX, and 40 μM Nec‐1 [Medchemexpress]; synoviocytes were challenged with 40 μM Nec‐1 for 1 hour before induction by TNF‐α + CHX). After 6 h, the conditioned media (CM) in N, TC, and TCN groups were centrifugated (1000 g, 5 min), collected and stored (−80°C).

As described previously,^25^ the chondrocytes were prepared from condylar fracture tissue, cultured into passage 3, then seeded into plates. Before treatment, TMJ synoviocytes‐CM (N‐CM, TC‐CM, or TCN‐CM) were diluted at 1:1 with medium. Gene expression was analysed based on samples harvested at 6, 12 or 24 h of treatment.

### 2.7 Cell cycle progression analysis

TMJ synoviocytes were exposed to TNF‐α (10 ng/mL), CHX (10 μg/mL) and Nec‐1(40 μM) for 12 h as mentioned above. Synoviocytes were rinsed with PBS and fixed in 70% cold ethanol for 24 h at 4°C. After washing synoviocytes with PBS, they were removed and stained with propidium iodide (PI), and cell cycle analysis was performed by employing a FACS Calibur device (BD Biosciences) and CellQuest software.

### 2.8 Immunofluorescence

Introduction of TNF‐α and CHX caused the translocation of NF‐κB from the cytoplasm to the nucleus. Synoviocytes were pretreated with Nec‐1 (40 μM) for 1 h and then stimulated with TNF‐α (10 ng/mL) and CHX (10 μg/mL) for 6 h. After fixation with 4% paraformaldehyde, synoviocytes were treated with goat serum for 1 h and then incubated with NF‐κB‐p65 antibody (1:100; Zen Bioscience) overnight. Synoviocytes were then treated with Alexa Fluor 555‐conjugated goat anti‐rabbit antibody (1:400; BIOSS) for 1 h, stained with DAPI and photographed using an Olympus microscope (Olympus).

### 2.9 Western blot

Protein was isolated from TMJ synoviocytes and measured by the Enhanced BCA Protein Assay Kit (Beyotime). Cell lysates (20 μL per sample) were first added to the samples, which were subsequently sorted using 10% SDS‐PAGE. Proteins were treated overnight with the specified antibodies, including RIPK1 (1:500; Proteintech), cell inhibition of apoptosis protein (cIAP) (1:5000; Proteintech), p‐p65 (1:500; Zen Bioscience), p‐p38 (1:500; Zen Bioscience), p‐RIPK1 (1:1000; CST) and RIPK3 (1:1000; CST). (1:1000; Bioss). The loading control was set by blotting GAPDH (1:2000; Bioss). Images were captured and examined using Image J.

### 2.10 Flow cytometry

Annexin V‐FITC/PI kit was employed to assess the apoptotic of synoviocytes challenged with TNF‐α (10 ng/mL) and CHX (10 μg/mL) with or without Nec‐1 (40 μM). After harvesting synoviocytes, they were washed with pre‐cooled PBS and mixed in a binding buffer. The mixing buffer was then supplemented with PI (5 L) and Annexin V (5 L), and stored at 4°C for 15 min. The BD FACS Calibur (BD Biosciences) was utilized to characterize the apoptotic stages of synoviocytes at early and late stages.

### 2.11 Quantitative real‐time polymerase chain reaction (qRT‐PCR)

TMJ synoviocytes were lysed using RNAiso Plus (Takara). To obtain cDNA, total RNA was extracted and reversely transcribed by PrimeScriptTM RT kit and gDNA Eraser (Takara). A total of 40 cycles of qRT‐PCR was then run on the BIORAD real‐time PCR system (CFXConnect) and Power TB Green PCR Master Mix (Takara). GAPDH is the standard. The 2^−ΔΔct^ technique was employed to equalize the level of the test gene to the level of GAPDH. Table S1 presents the primer sequences.

### 2.12 Statistical analysis

Experimental data were analysed and plotted using Graphpad Prism 8 software (Graphpad Prism). Data are presented as mean ± S.D. Multiple comparisons were made using one‐way anova and independent samples *t*‐tests were used to compare two groups.

## 3 RESULTS

### 3.1 Inhibiting RIPK1 shows therapeutic effects on the synovium and cartilage in rat TMJOA models

**FIGURE 1:**
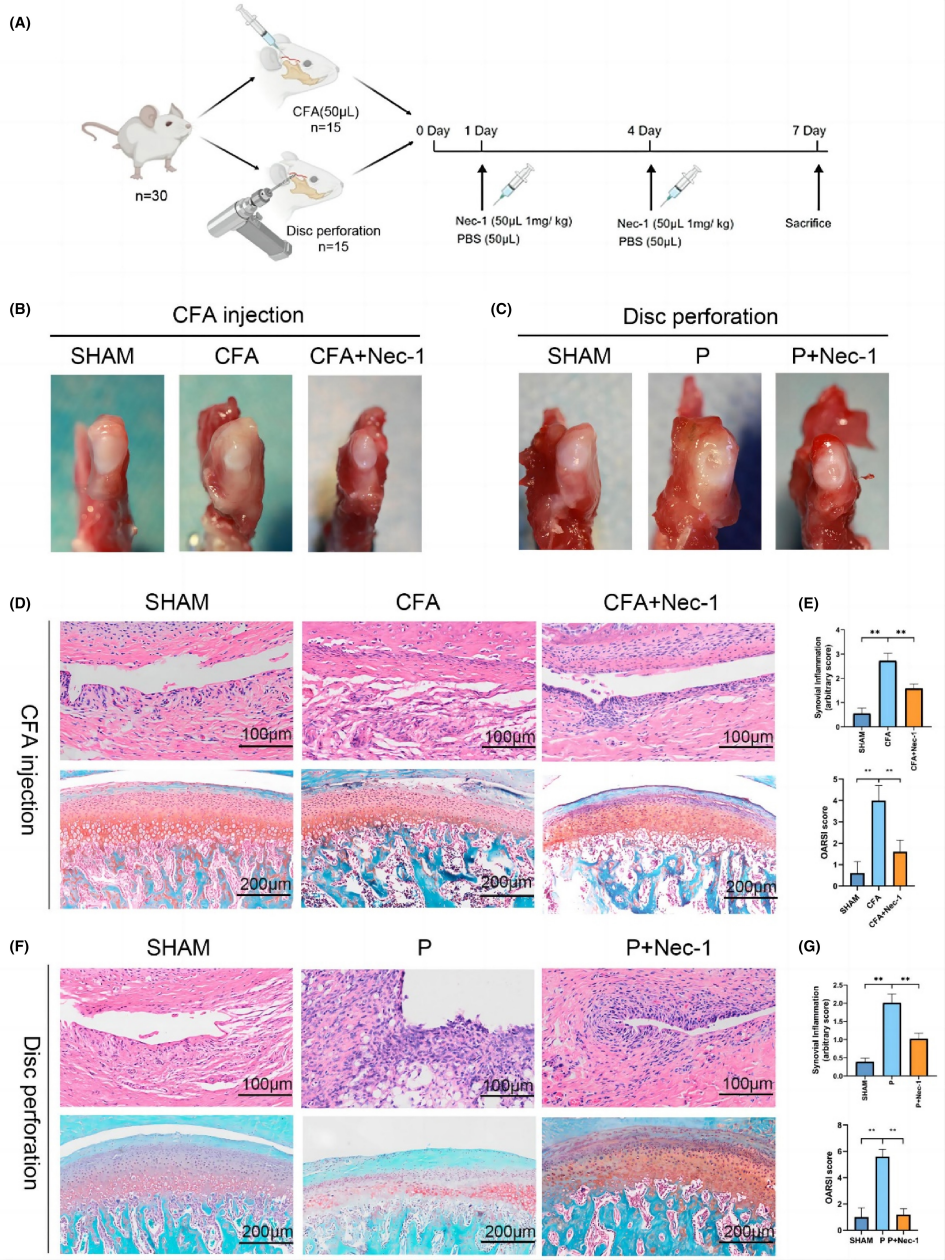
Inhibiting RIPK1 alleviates CFA‐induced/disc‐perforation‐induced TMJOA. (A) Outline of in‐vivo design. (B) Morphological characteristics of peri‐condylar in the CFA‐induced TMJOA model. (C) Morphological characteristics of peri‐condylar in the disc‐perforation‐induced TMJOA model. (D) HE staining of the synovium and Safranin O and fast green staining of cartilage in the CFA‐induced TMJOA model. (E) Synovial inflammation scores (*n* = 10, scale bar = 100 μm), OARSI grades (*n* = 10, scale bar = 200 μm), in the CFA‐induced TMJOA model. (F) HE staining of synovium and Safranin O and fast green staining of cartilage in the disc‐perforation‐induced TMJOA model. (G) Synovial inflammation scores (*n* = 10, scale bar = 100 μm), OARSI grades (*n* = 10, scale bar = 200 μm), in the disc‐perforation‐induced TMJOA model. SHAM: sham‐operation group. CFA, CFA‐induced model group. TMJOA, temporomandibular joint osteoarthritis. P, disc‐perforation‐induced model group. Nec‐1, treatment group. *, *p* < 0.05; **, *p* < 0.001.

To assess the curative effects of inhibiting RIPK1 in vivo, SD rat models of CFA‐induced and disc‐perforation‐induced TMJOA were established (Figure 1A). After treatment with Nec‐1, the degree of swelling and hyperplasia in the perichondral tissue of TMJ was less pronounced than that in the CFA/P group (Figure 1B,C). H&E staining revealed that the CFA/P group had more lymphocyte infiltration in the synovium than the other two groups. However, after Nec‐1 treatment, fewer inflammatory cells were recruited (Figure 1D,F). In addition, the CFA/P group presented more severe synovial inflammation than the sham group (Figure 1E,G).

The histopathology of cartilage was assessed by the modified OARSI grading system. The Nec‐1 treatment group had a lower OARSI grade than the CFA/P group (Figure 1E,G). The CFA/P group demonstrated heavier cartilage erosion and proteoglycan loss. However, the CFA/P + Nec‐1 group showed smoother cartilage and milder proteoglycan loss than other groups (Figure 1D,F).

**FIGURE 2:**
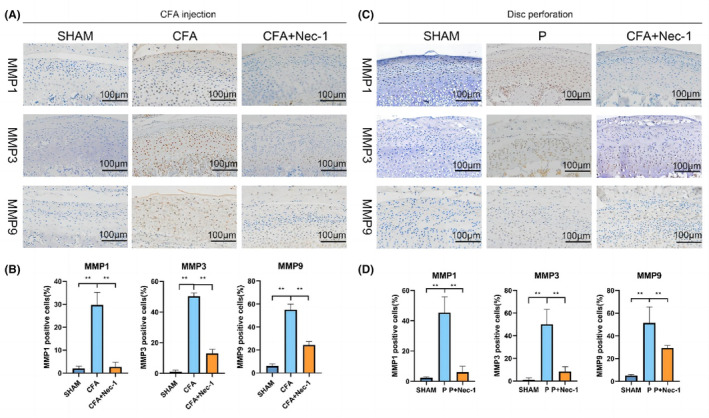
Inhibiting RIPK1 has chondroprotective effects on the TMJ. (A) Immunohistochemical analysis of MMPs in the cartilage in CFA‐induced TMJOA. (B) Quantification analysis of (A). (C) Immunohistochemical analysis of MMPs in the cartilage in disc‐perforation‐induced TMJOA. (D) Quantification analysis of (C). SHAM: sham‐operation group. CFA, CFA‐induced model group. MMP, matrix metalloproteinases; TMJOA, temporomandibular joint osteoarthritis; P, disc‐perforation‐induced model group. Nec‐1, treatment group. *, *p* < 0.05; **, *p* < 0.001.

Nec‐1 brought a significant cartilage‐protective effect in the CFA‐induced inflammation model as the expression of MMP1, MMP3 and MMP‐9 dropped (Figure 2A,B). Immunohistochemistry also revealed higher protein expression of MMP1, MMP3, and MMP‐9 in the P group than in the SHAM group, which were all reduced in the P + Nec‐1 group (Figure 2C,D).

### 3.2 Nec‐1 exerts no effect on the cell cycle and proliferation but maintains the activity of TMJ synoviocytes co‐stimulated by TNF‐α and CHX

**FIGURE 3:**
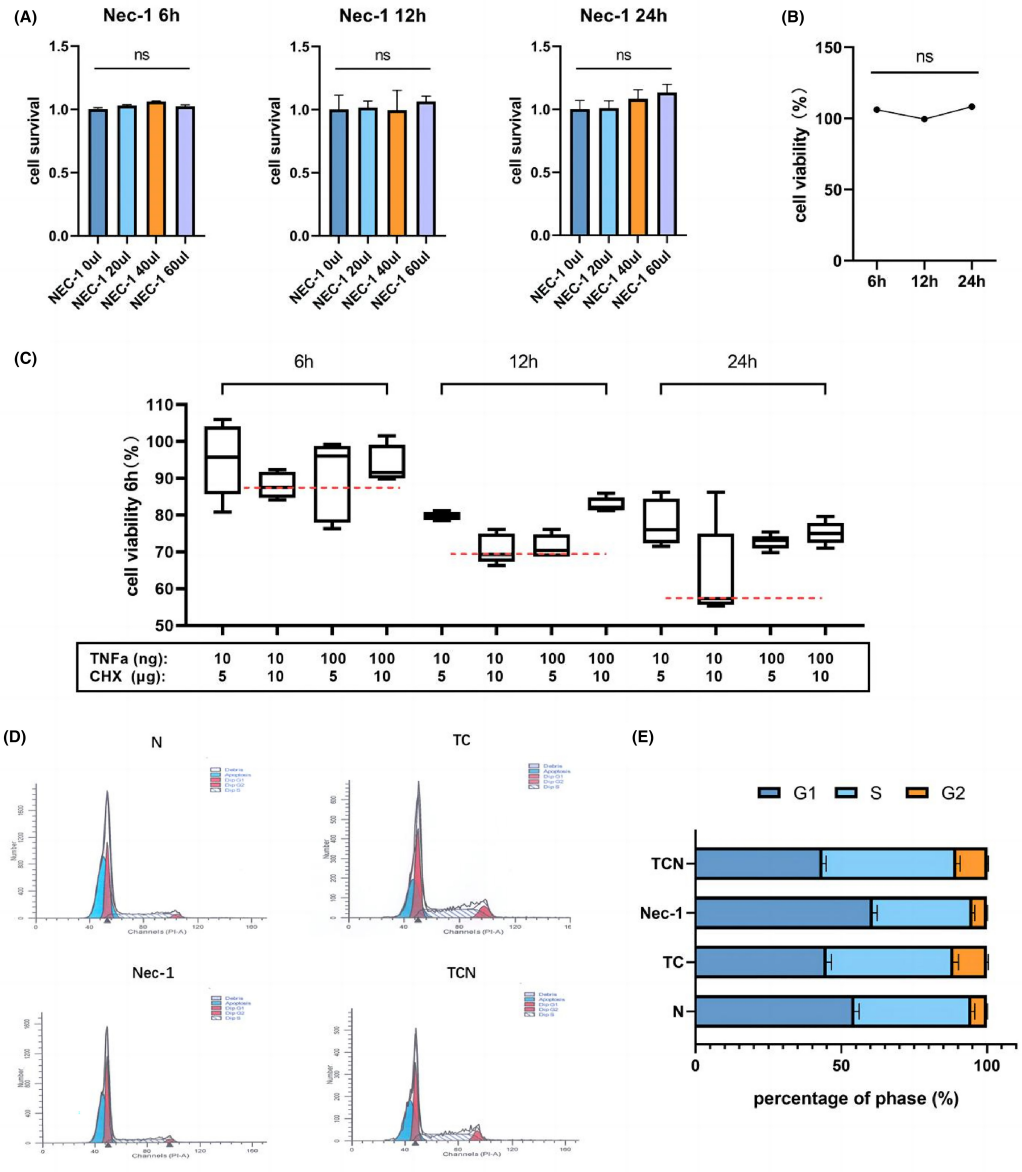
Nec‐1 exerts no effect on the cell cycle and proliferation. (A) Synoviocytes were incubated in culture medium containing Nec‐1 (20, 40 and 60 μM) for 6, 12 and 24 h. (B) Synoviocytes were incubated in culture medium containing 40 μM Nec‐1 for 6, 12 and 24 h; (C) Synoviocytes were incubated in several concentrations of TNF‐α and CHX for 6, 12 and 24 h. (D) Flow cytometric cycle analysis. (E) Quantitative analysis of (D). N, control, TC, synoviocytes treated with TNF‐α and CHX, Nec‐1, synoviocytes treated with Nec‐1, TCN, synoviocytes treated with TNF‐α, CHX, and Nec‐1. ns, no significant, *, *p* < 0.05; **, *p* < 0.001.

Nec‐1 (0, 20, 40 and 60 μM) did not decrease the viability or proliferation of TMJ synoviocytes at 6, 12 or 24 h (Figure 3A,B). So, the Nec‐1 concentration was set at 40 μM to maintain cell viability in the following in vitro experiments.^26^

Co‐stimulation with TNF and CHX reduced cell viability at a combination of concentrations (Figure 3C). When 10 ng/mL TNF and 10 μg/mL CHX were co‐stimulated, the strongest effect on lowering cell viability was reported in these groups.

As shown in Figure 3B, 40 μM Nec‐1 did not inhibit the proliferative viability of TMJ synoviocytes, and cell cycle analysis confirmed this finding (Figure 3D). Figure 3E includes a percentage analysis of cell cycle phases, which demonstrates that Nec‐1 did not affect synoviocyte proliferation.

### 3.3 TMJ synoviocytes‐cultured medium interferes with inflammatory responses in chondrocytes

**FIGURE 4:**
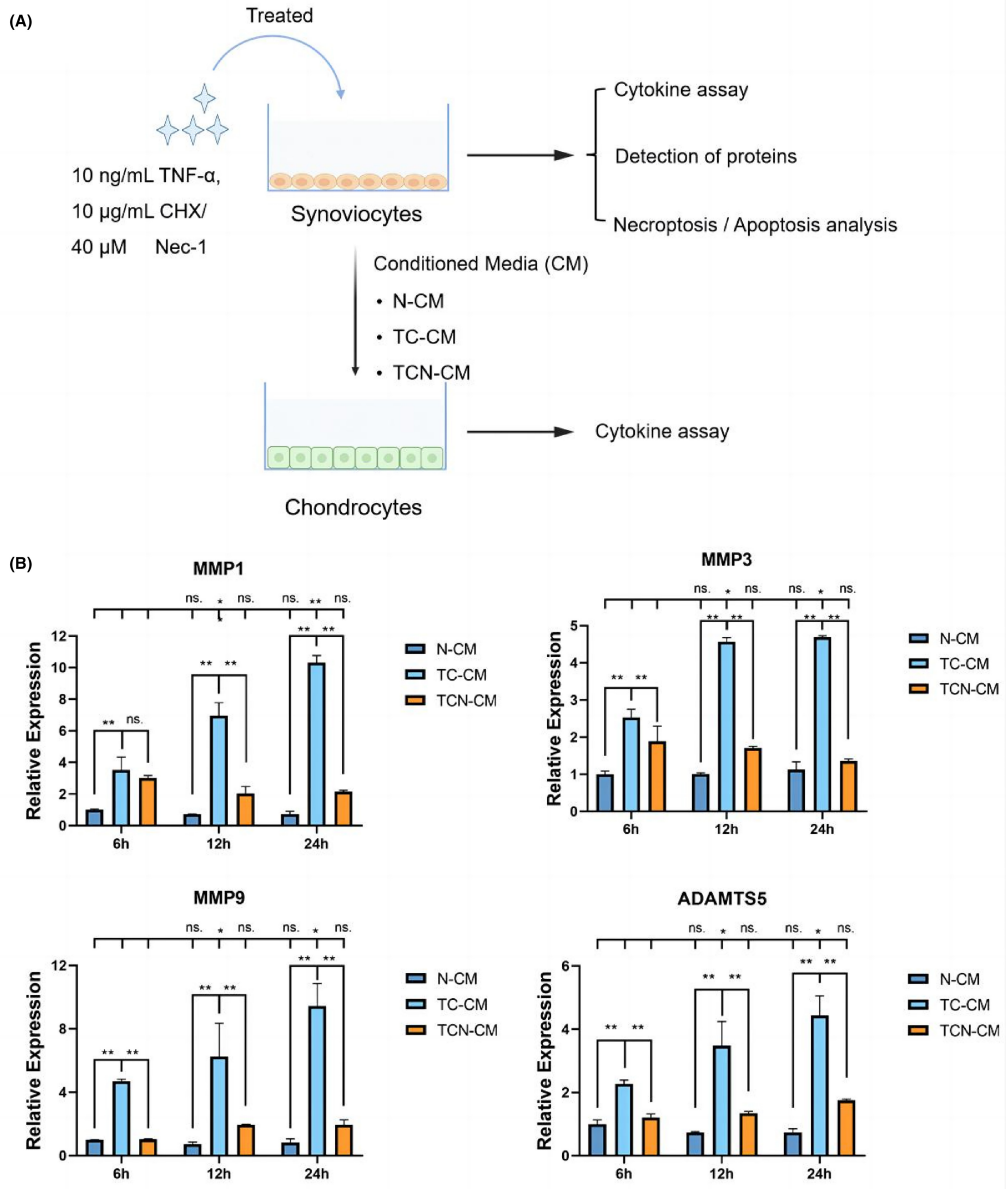
TMJ synoviocytes‐conditioned medium regulates inflammatory reactions in chondrocytes. (A) In vitro experimental procedures and sample assignment. (B) The mRNA expression of MMPs in chondrocytes, N‐CM, normal synoviocytes‐derived CM, TC‐CM, synoviocytes treated by TNF‐α and CHX‐derived CM, TCN‐CM: synoviocytes treated by TNF‐α, CHX, and Nec‐1‐derived CM. MMP, matrix metalloproteinases; TMJ, temporomandibular joint; ns, no significant, *, *p* < 0.05; **, *p* < 0.001.

To determine whether synoviocytes interact with chondrocytes, normal synoviocytes‐derived CM (N‐CM), synoviocytes induced with TNF‐α and CHX‐derived CM (TC‐CM), or Nec‐1‐derived CM (TCN‐CM) were used to stimulate chondrocytes (Figure 4A). The results showed that MMP1, MMP3, MMP9 and ADAMTs5 appeared to be highly expressed in chondrocytes treated with TC‐CM. In contrast, TCN‐CM significantly reversed this high expression (Figure 4B).

### 3.4 Inhibiting RIPK1 suppresses TNF‐α‐induced inflammatory gene expression in TMJ synoviocytes and RIPK1 regulates NF‐κB activation via cIAP

**FIGURE 5:**
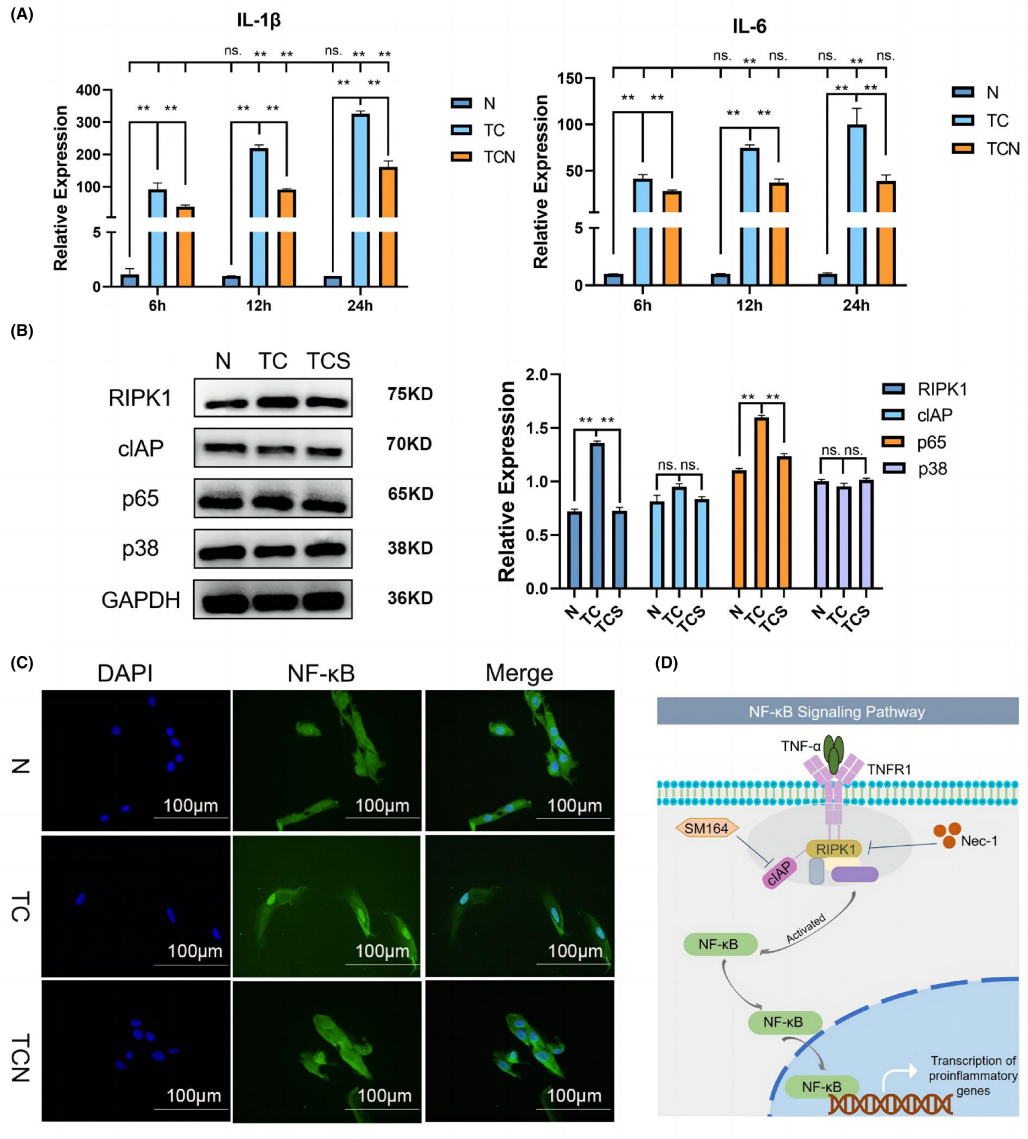
Inhibiting RIPK1 suppresses TNF‐α induced inflammatory progression in synoviocytes. RIPK1 regulates NF‐κB activation via cIAP. (A) mRNA of IL‐1β and IL‐6 in synoviocytes. (B) Protein expression of RIPK1, cIAP, p65, and p38 in synoviocytes, and their semi‐quantitative analysis. (C) Immunofluorescence of NF‐κB p65 transfer. (D) Schematic diagram of RIPK1 regulation of TMJOA via NF‐κB. N: control, TC: synoviocytes treated with TNF‐α and CHX, TCN: synoviocytes treated with TNF‐α, CHX, and Nec‐1, synoviocytes were pretreated with Nec‐1 for 1 h before treatment with TNF‐α and CHX, TCS, synoviocytes treated with TNF‐α, CHX, and SM164, synoviocytes were pretreated with SM164 for 2 h before treatment with TNF‐α and CHX. ns, no significant, *, *p* < 0.05; **, *p* < 0.001.

Pre‐treatment of TMJ synoviocytes with Nec‐1 for 1 h before co‐stimulation with TNF‐α and CHX (Figure 4A). Synoviocytes secreted higher levels of IL‐1β and IL‐6 after TNF and CHX stimulation. However, pretreatment with Nec‐1 notably lowered levels of the two factors (Figure 5A).

The effect of RIPK1 on the MAPK and NF‐κB was investigated. As shown in Figure 5B, TNF‐α and CHX stimulation increased RIPK1, cIAP and p‐p65 levels. But in SM164 (cIAP inhibitor) ‐pretreated cells, RIPK1, and p‐p65 levels fell significantly. However, p‐p38 and cIAP levels did not change after any of these treatments.

Immunofluorescence was used to explore whether NF‐κB p65 was activated. TNF‐α and CHX induced the transfer of NF‐κB p65 from the cytoplasm into the cell nucleus. Interestingly, pretreatment with Nec‐1 impeded this translocation (Figure 5C). The above results suggest that RIPK1 can activate synovial inflammatory pathways by driving NF‐kB into the nucleus via cIAP (Figure 5D).

### 3.5 RIPK1 works with caspase‐8 to regulate the apoptosis and the inflammatory progression in TMJOA

**FIGURE 6:**
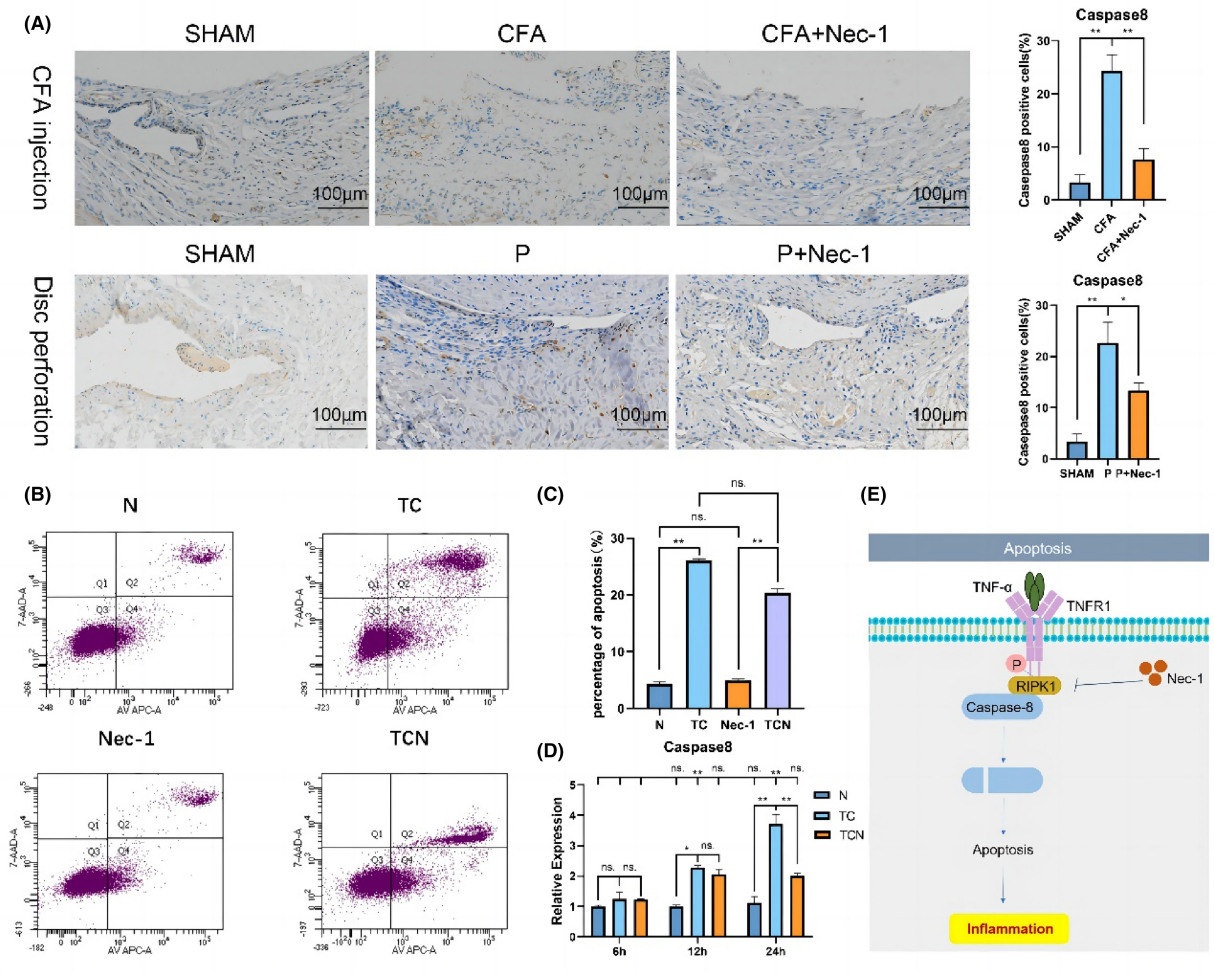
Inhibiting RIPK1 protects against TNF‐α‐induced apoptosis. RIPK1 regulates apoptosis through caspase‐8. (A) Immunohistochemical analysis of Caspase‐8 in the synovium in the CFA‐induced and disc‐perforation‐induced TMJOA models. (B) Flow cytometry results of synoviocytes. (C) Analysis of synoviocytes apoptosis rates. (D) The mRNA expression of Caspase‐8 in synoviocytes. (E) Schematic diagram of RIPK1 regulation of TMJOA via caspase‐8. SHAM, sham‐operation group; CFA, CFA‐induced model group; P, disc‐perforation‐induced model group; Nec‐1, treatment group. *, *p* < 0.05; **, *p* < 0.001. N, control; TC, cells treated with TNF‐α and CHX; Nec‐1, cells treated with Nec‐1; TCN, cells treated with TNF‐α, CHX, and Nec‐1; cells were pretreated with Nec‐1 for 1 h before treatment with TNF‐α and CHX. ns, no significant, *, *p* < 0.05; **, *p* < 0.001.

We investigated the regulating mechanism of RIPK1 in TMJOA. Immunohistochemical analysis of synovial tissues from two rat TMJOA models revealed that the expression of caspase‐8, an apoptotic marker, was considerably higher in both CFA/P groups compared to the SHAM group but inverted by Nec‐1 (Figure 6A).

Compared to the TNF‐α and CHX group, Annexin V‐FITC/PI staining showed a lower level of apoptosis in synoviocytes in the Nec‐1 treated group (Figure 6B, C). Correspondingly, Caspase‐8 expression was significantly augmented in TNF‐α‐induced synoviocytes, then reversed by Nec‐1 (Figure 6D).

These observations implicate that RIPK1 in inducing apoptosis by interacting with caspase‐8 and thereby influences the evolution of TNF‐induced inflammation in TMJOA (Figure 6E).

### 3.6 RIPK1 influences TMJOA inflammatory development by activating synoviocytes necrosis signalling pathway through interactions with RIPK3, and MLKL

**FIGURE 7:**
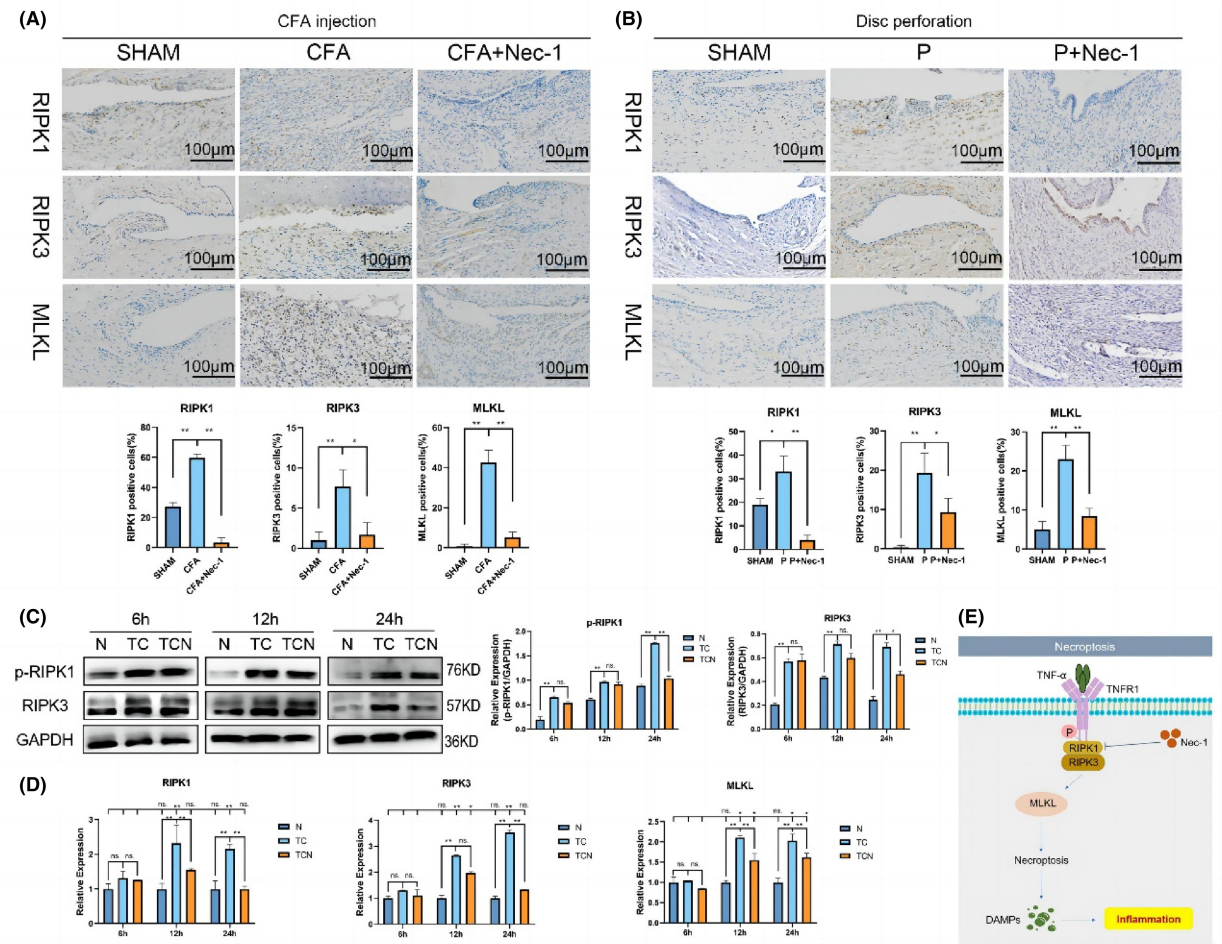
Inhibiting RIPK1 protects against TNF‐α‐induced necroptosis. RIPK1 regulates necrosis through RIPK3/MLKL. (A) Immunohistochemical analysis of RIPK1/RIPK3/MLKL in synovium in CFA‐induced TMJOA. (B) Immunohistochemical analysis of RIPK1/RIPK3/MLKL in synovium in disc‐perforation‐induced TMJOA. (C) Western blot for protein expression levels of p‐RIPK1 and RIPK3, and their semi‐quantitative analysis. (D) The mRNA levels of RIPK1, RIPK3 and MLKL in synoviocytes. (E) Schematic diagram of RIPK1 regulation of TMJOA via RIPK3/MLKL. SHAM: sham‐operation group. P: disc perforation model group. Nec‐1, treatment group; N, control; TC, cells treated with TNF‐α and CHX; TCN, cells treated with TNF‐α, CHX, and Nec‐1; cells were pretreated with Nec‐1 for 1 h before treatment with TNF‐α and CHX. ns, no significant, *, *p* < 0.05; **, *p* < 0.001.

The level of RIPK1/RIPK3/MLKL expression was upregulated in the CFA/P group compared to the control group, whereas it was downregulated in the Nec‐1‐treated group (Figure 7A,B).

The phosphorylation of PIPK1 and RIPK3, two typical markers of necroptosis, was assessed in vitro. Western blot demonstrated that TNF‐α and CHX resulted in protein elevation of p‐RIPK1 and RIPK3, which was reduced by Nec‐1 (Figure 7C).

TNF‐α and CHX co‐stimulation markedly elevated the mRNA levels of RIPK1, RIPK3 and MLKL (Figure 7D). As expected, the inflammation triggered by the RIPK1‐RIPK3 is mediated by necrosis effector protein MLKL.

The above results show that RIPK1 affects TMJOA inflammatory progression by cooperating with RIPK3 and MLKL to activate the synoviocyte necrosis signalling pathway (Figure 7E).

## 4 DISCUSSION

In the current research, we found that inhibition of RIPK1 could counteract TMJ cartilage inflammation by inhibiting apoptosis, necrosis and NF‐κB activity in TMJ synoviocytes. TMJOA features pain, dental malocclusion, and limited mouth opening, always ending up with synovial inflammation, cartilage degeneration and subchondral bone reconstruction.^27^, ^28^ RIPK1 has been implicated in the pathophysiology of a number of degenerative illnesses, such as amyotrophic lateral sclerosis, as well as neurodegenerative and inflammatory diseases.^21^ RIPK1 has a unique hydrophobic pocket between the N‐ and C‐terminal ends of the kinase that regulates kinase activation through allosteric modulation.^21^ Nec‐1 binds to this hydrophobic pocket and stabilizes RIPK1 in an inactive state. Nec‐1 can specifically inhibit the phosphorylation of RIPK1. It has been reported the effect of Nec‐1 on pathological cartilage thinning is caused by mechanical force.^29^ However, the regulatory mechanism of RIPK1 in TMJOA has not been explored. Our findings uncovered that inhibition of RIPK1 suppresses synovitis to alleviate TMJOA.

TNF‐α stimulates cells to produce inflammatory factors or induces cell death.^30^ TNF‐α binds to TNFR1 and recruits RIPK1 to form complex I. Simultaneously, cIAPs are also recruited to complex I. Reciprocally, cIAPs ubiquitylate the components inside the complex I.^31^ Complex I activates the NF‐κB and MAPK pathways, resulting in the translocation of NF‐κB from the cytoplasm to the nucleus, which can lead to increased expression of inflammatory genes.^32^ However, cIAPs fail to ubiquitinate RIPK1, and the complex IIa builds up to activate caspase‐3, thus initiating the process of apoptosis procedure.^33^ In addition to activating apoptotic mechanisms, RIPK1 induces the expression of inflammatory cytokines and chemokines in apoptotic cells. The combined action of apoptosis and inflammatory cytokines/chemokines may be particularly effective in recruiting and activating inflammatory cells and triggering inflammatory progression. Once caspase‐8 is repressed, RIPK1 and RIPK3 associate to form complex IIb, which enhances the phosphorylation of RIPK3. Once activated, RIPK3 then induces the phosphorylation of MLKL (p‐MLKL). As p‐MLKL migrates to destroy the cell wall, necroptosis starts.^34^ Necroptosis, always emerging with the spillover of endogenous ligands for damage‐associated molecular patterns (DAMPs), can plunge innate immune cells into inflammatory responses.

In the condition of inflammation, synoviocytes are driven to secrete more proinflammatory cytokines. Meanwhile, the release of aggrecan and collagen II is suppressed, followed by the overproduction of MMP1, MMP3, MMP9 and ADAMTs5, all as key regulators of cartilage destruction.^35^, ^36^ RIPK1 activity can be triggered by co‐stimulation of TNF‐α and CHX. CHX also mediates the sensitization of chondrocytes to TNF‐induced apoptosis.^37^ More necrosis and apoptosis markers are produced in the synovium of osteoarthritis mice than in normal mice.^38^, ^39^ In the present in vitro experiments, we tested the viability of synoviocytes at three‐time points, finding that continuous exposure to TNF and CHX at a certain concentration can realize appropriate induction of necroptosis.

The dose of Nec‐1 should be set with caution, because a high dose may bring about cytotoxicity. Nec‐1 has shown its cytoprotective effect in ischemic brain damage,^40^ cardiac ischemia,^41^ anaplastic thyroid, and adrenocortical cancers.^42^ In a cell experiment, Nec‐1 was dosed at 10 to 300 μM.^43^, ^44^, ^45^, ^46^ Most concentrations of Nec‐1 used in similar cellular experiments in the literature are 40 μM, and these experiments have shown that this concentration of Nec‐1 significantly reduces the number of apoptotic and necrotic cells.^45^, ^46^ Here we set the dose of Nec‐1 at 40 μM in vitro, which proved that Nec‐1 at 40 μM did not reduce cell activity. Hence, to control the aspects of the cytotoxic drug on synoviocytes, we performed assays to evaluate cellular activity, proliferation and apoptosis. We found that synoviocytes could maintain their viability and growth at certain Nec‐1 concentrations.

Inhibiting RIPK1 can effectively curb the progression of joint inflammation.^47^ It has been shown that the level of TNF‐α‐induced inflammatory mediators falls in human TMJ synoviocytes treated with Nec‐1. Consistently, our results showed that TNF‐α and CHX‐derived CM (TC‐CM) elevated the expression of inflammatory factors (e.g. MMPs, and ADAMTS‐5.) in human TMJ chondrocytes, but Nec‐1‐derived CM (TCN‐CM) reduced the expression of inflammatory factor, indicating that inhibition of RIPK1 can attenuate chondrocyte inflammation by keeping synoviocytes from secreting inflammatory factor in TMJ.

RIPK1 is an important administrator in the TNF‐α/TNFR1‐ NF‐κB signalling. TNF‐α activates the cascade involving NF‐κB or terminal MAPK, extracellular signal‐regulated kinase (ERK) and Jun N‐terminal kinase (JNK).^48^ NF‐κB and MAPK participate in signalling pathways responsible for MMPs production and chondrocyte proliferation and differentiation. It was shown that NF‐κB and MAPK collaborate to adversely regulate RIPK1 biological activity.^19^ cIAPs, suppressed by SM‐164, mediates both the NF‐κB and RIPK1 activity in fibroblasts.^49^ In the present study, inhibiting cIAPs suppressed NF‐κB signalling, as indicated by the low level of p65. However, the change in p38 phosphorylation was not significant, which is inconsistent with the previous studies.^50^

Nec‐1 can allosterically block RIPK1‐dependent NF‐κB activation, though how RIPK1 activates NF‐κB remains unclear. It seems that RIPK1 activation is not necessary for the expression of NF‐κB‐mediated inflammatory cytokines. However, our results showed that Nec‐1 quells p65 phosphorylation, which may explain the therapeutic effects of Nec‐1 on TMJOA. Nec‐1 can inhibit the activity of NF‐κB in OA.^51^ Hence, RIPK1 can be activated to promote NF‐κB‐mediated inflammatory cytokine expression in a certain context. The interaction between complex regulatory pathways in TMJOA should be elucidated with further research.

We also verified that RIPK1 could be downregulated to suppress TNF‐α‐derived apoptosis in synoviocytes. Apoptosis is a culprit of either cartilage destruction or matrix catabolism in OA tissue.^52^ OA aggravates as inflammatory mediators are released by apoptotic cells.^53^ The autophosphorylation of RIPK1 changes its conformation, and caspase‐8 leads to the cleavage of caspase‐3/7 to initiate cell apoptosis.^54^ Blocking caspase‐8 activation can lead to the oligomerization and auto‐phosphorylation of RIPK1 and RIPK3.^55^ Therefore, caspase‐8 can mark the process of apoptosis. In the present study, inhibiting RIPK1 counteracted TNF‐α‐activated apoptosis and inflammation in synoviocytes. In addition, apoptosis was less pronounced in the TNF‐α, CHX, and Nec‐1‐combined treatment group. These results suggest that RIPK1 regulates synoviocyte apoptosis via caspase‐8, whose kinase activity can be repressed to pose anti‐apoptotic and anti‐inflammatory effects on TMJOA in vitro.

RIPK1 also regulated necroptosis in synoviocytes induced by TNF‐α. Necroptosis is a new process that underpins programmed cell death. During necroptosis, S166 is phosphorylated in the kinase structure of RIPK1.^56^ Increased phosphorylation of S166 (RIPK1) was detected after TNF and CHX stimulation in this study. Phosphorylated RIPK1 can bind to RIPK3, inducing the development of the necrosome complex.^55^ In this complex, MLKL phosphorylation ensuing RIPK3 autophosphorylation enables MLKL to translocate from cytoplasm to membrane.^57^ Earlier, researchers demonstrated that TNF‐α and CHX could cause necroptosis in vitro. Indeed, necroptosis still predominates despite the competitiveness of apoptotic pathways.^58^ In the current research, inhibiting RIPK1 reduced protein expression levels of necrosis markers, compared to those under inflammatory stimulation, suggesting that RIPK1 affects TMJOA progression by regulating synovial cell necrosis through RIPK3 and MLKL.

Above mentioned findings were validated in rat models of CFA and disc perforation‐induced TMJOA. TMJOA is a multifactorial disease, usually caused by inflammation or structural disorder (disc displacement or disc perforation). TMJOA can be induced in different animal models. In our study, the inflammatory response was caused by an injection of CFA into the upper chamber of TMJ. CFA injection causes rapid death of many chondrocytes and synoviocytes, resulting in joint injury and pain.^59^ Therefore, intra‐articular injection is usually used in animal models to research into molecular mechanisms of inflammation in osteoarthritis and search for preclinical treatments.^60^ To explore the effect of the RIPK1 inhibitor on structural disorder‐induced TMJOA, we also established a model in which surgery was performed to damage the structure and provide abnormal articular forces to induce OA‐like lesions. Disc perforation, as one induction surgery, well mimics advanced symptoms of TMJOA induced by structural disorder in clinical patients. In our models, we also found that CFA and disc perforation‐induced inflammation triggered synovial hyperplasia and inflammatory infiltration, which were then alleviated by Nec‐1. In addition, immunohistochemistry indicated that Nec‐1 lowered the protein levels of necroptosis markers (RIPK1/RIPK3/MLKL) and the apoptosis marker (Caspase‐8). Furthermore, RIPK1 acts synergistically in necrosis and apoptosis, implicating RIPK1 upstream of pathways associated with both. These findings demonstrate that necroptosis and apoptosis cooperate in the pathogenesis of TMJOA.

In conclusion, inhibiting RIPK1 can fight against inflammation in synoviocytes, the mechanism of which involves the NF‐κB, apoptosis and necrosis pathways. RIPK1 regulates NF‐κB activation via cIAPs, apoptosis via caspase‐8, and necroptosis via RIPK3 and MLKL in synoviocytes, all of which affect TMJOA inflammation progression (Figure SS1). RIPK1 can be a promising target for new treatment in TMJOA.

## AUTHOR CONTRIBUTIONS

**Xin Cao:** Data curation (lead); formal analysis (lead); methodology (lead); writing – original draft (lead); writing – review and editing (lead). **Sisi Peng:** Data curation (supporting); formal analysis (supporting). **Ying Yan:** Data curation (equal); formal analysis (equal). **Jun Li:** Formal analysis (equal); funding acquisition (equal). **Jianping Zhou:** Formal analysis (equal); funding acquisition (equal). **Hongwei Dai:** Resources (equal); validation (equal); writing – review and editing (equal). **Jie Xu:** Funding acquisition (equal); resources (equal); writing – original draft (equal); writing – review and editing (equal).

## CONFLICT OF INTEREST STATEMENT

The authors declare that they have no competing interests.

## ETHICS STATEMENT

The research was allowed by the Committee of Chongqing Medical University (CQHS‐REC‐2021 [LSNo27]). Before the study began, each participant completed a declaration of consent. The synovial tissue and cartilage were sampled according to the Declaration of Helsinki.

## Supporting information


Figure S1:

Table S1

## Data Availability

The data that support the findings of this study are available on request from the corresponding author.
