# Supplementary figures and images for: Alleviation of temporomandibular joint osteoarthritis by targeting RIPK1‐mediated inflammatory signalling
Source: J Cell Mol Med. 2023 Aug 29;28(5):e17929. doi: 10.1111/jcmm.17929 (PMC10902568; doi:10.1111/jcmm.17929)

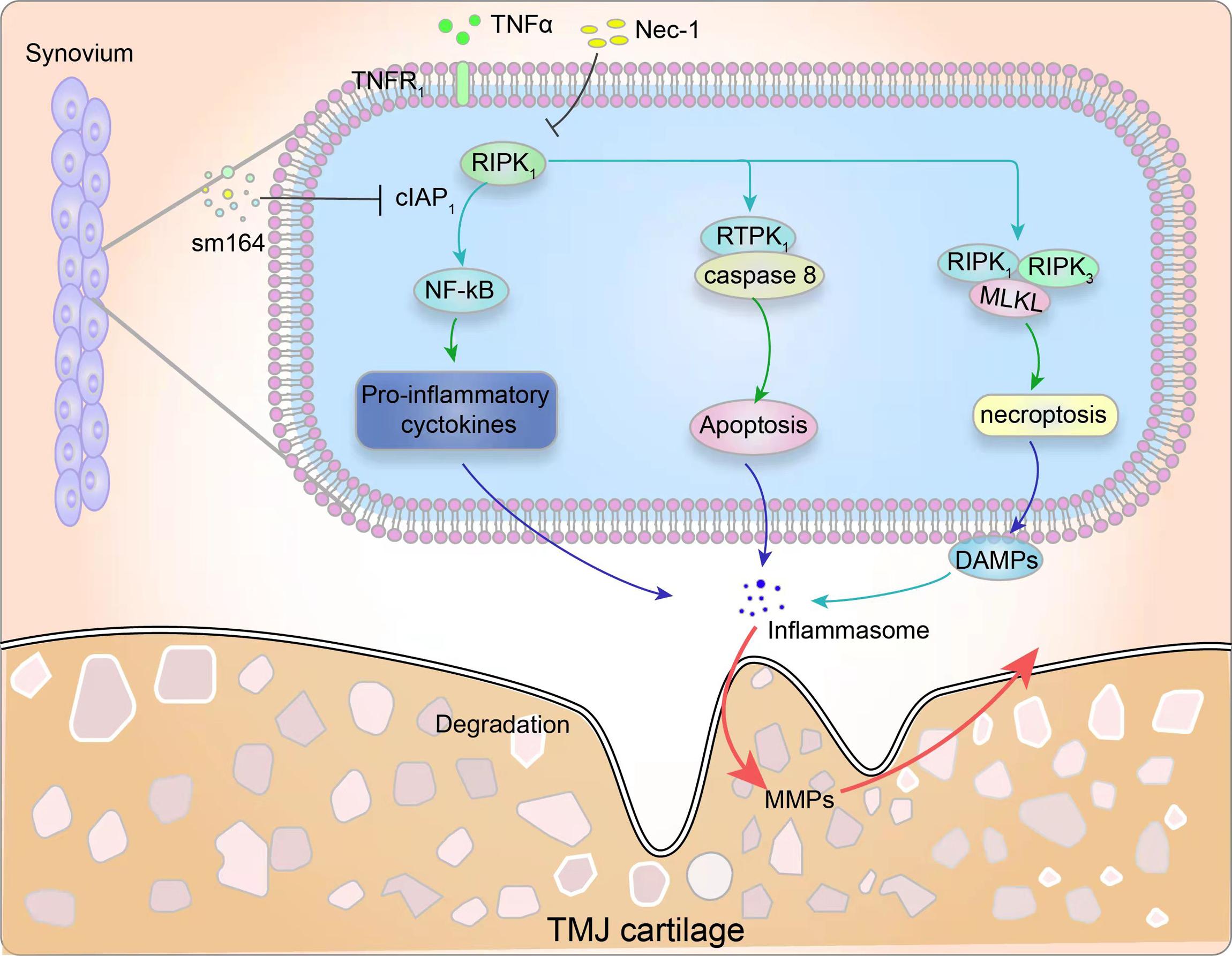

Supplement: Supplementary file 1 — Figure S1: [file JCMM-28-e17929-s001.jpg]
