# Supplementary material for: Alleviation of temporomandibular joint osteoarthritis by targeting RIPK1‐mediated inflammatory signalling
Source: J Cell Mol Med. 2023 Aug 29;28(5):e17929. doi: 10.1111/jcmm.17929 (PMC10902568; doi:10.1111/jcmm.17929)
Supplement: Supplementary file 2 — Table S1 [file JCMM-28-e17929-s002.docx]

Supplementary Table 1 Human (h) qRT‐PCR primers used in this study

| Gene | Primer sequence (forward) | Primer sequence (reverse) |
| --- | --- | --- |
| hRIPK1 | TATGGAGATTGGTGGGACGAG | TGGGTCCAGGTGTTTATCCG |
| hRIPK3 | CCTGCTGAAAGAAGTGGTGC | CTGTGAGCCTCCCTGAAATG |
| hMLKL | CTGCCCTGGAGGAGGCTAAT | ATGCGTTGCTCAACCTGAAGTA |
| hCaspase8 | GCAAACTGGATGATGACATGAAC | AGCAGGCTCTTGTTGATTTGG |
| hIL‐1β | AATCTGTACCTGTCCTGCGTGTT | TGGGTAATTTTTGGGATCTACACTCT |
| hIL‐6 | AGCCCACCGGGAACGA | GGACCGAAGGCGCTTGT |
| hMMP1 | ACTGCCAAATGGGCTTGAAG | TTCCCTTTGAAAAACCGGACTT |
| hMMP3 | GAGGCATCCACACCCTAGGTT | TCAGAAATGGCTGCATCGATT |
| hMMP9 | CCCTTGTGCTCTTCCCTGGA | TCTGCCACCCGAGTGTAACC |
| hADAMTS5 | GGCCTCCATCGCCAATAGG | GGATAGCTGCATCGTAGTGCT |
| hGAPDH | CTTTGGTATCGTGGAAGGACTC | GTAGAGGCAGGGATGATGTTCT |
